# Species- and endpoint-dependent phytotoxic responses of maize and lettuce to *Cinnamomum cassia* leaf aqueous extract: physiological, anatomical, and dose–response evidence

**DOI:** 10.3389/fpls.2026.1893917

**Published:** 2026-07-20

**Authors:** Wumei Si, Lexi Xie, Gang Xu, Quan Yang

**Affiliations:** School of Traditional Chinese Medicine, Guangdong Pharmaceutical University, Guangzhou, China

**Keywords:** allelopathic effect, *Cinnamomum cassia*, cinnamon leaf extract, dose-response, oxidative stress, phytotoxicity, root-tip meristem

## Abstract

Cinnamon leaf-derived materials have potential phytotoxic activity, but their effects may vary with concentration, recipient species, and biological endpoint. In this study, maize and lettuce were used as recipient plants to evaluate the laboratory-scale phytotoxic effects of *Cinnamomum cassia* leaf aqueous extract. A 100% stock solution was defined as 100 g dry leaf equivalent L⁻¹, and four treatments were established: 0%, 1%, 10%, and 20%, corresponding to 0, 1, 10, and 20 g dry leaf equivalent L⁻¹, respectively. Seed germination, seedling growth, physiological and biochemical traits, maize root-tip histology, correlation analysis, principal component analysis, and four-parameter logistic dose–response modeling were integrated to compare species- and endpoint-dependent responses. The extract showed concentration-dependent inhibitory effects on most growth traits, although low concentration treatment induced slight stimulation or compensatory responses in some indicators. Lettuce was more sensitive in germination and whole-seedling performance, whereas maize showed stronger inhibition in post-germination root elongation. High-concentration treatments were associated with increased MDA accumulation and altered SOD and POD activities, suggesting oxidative-stress-related responses and membrane lipid peroxidation. Maize root-tip sections showed reduced cell-row continuity and looser peripheral meristematic structure under high concentrations, and image-level ROI descriptors supported these anatomical observations. Based on relative root length, the preliminary model-derived EC50 values were 12.31% for lettuce and 5.61% for maize. These results indicate that cinnamon leaf aqueous extract induces species- and endpoint-dependent phytotoxic responses under laboratory conditions, and the observed physiological and anatomical changes are consistent with a possible pathway involving oxidative-stress-related imbalance, membrane damage, meristematic structural disturbance, and growth inhibition.

## 1 Introduction

Allelopathy is an ecological process in which plants release bioactive compounds that influence seed germination, seedling establishment, growth, and physiological metabolism of neighboring plants. This process is an important component of plant-plant interactions and provides a theoretical basis for the development of plant-derived weed-management materials (Rice, 1984; Inderjit and Duke, 2003; Cheng and Cheng, 2015; Khamare et al., 2022). Recent studies indicate that allelopathic effects are not limited to final germination percentage, but may involve multiple levels of plant response, including redox regulation, membrane lipid peroxidation, root chemical signaling, and changes in meristematic tissue organization (Kumar et al., 2024; Schulz and Tabaglio, 2024).

Cinnamon leaves are rich in phenylpropanoids, terpenoids, and other secondary metabolites, and cinnamon essential oils or extracts have been reported to inhibit seed germination and seedling growth in several recipient species (Cavalieri and Caporali, 2010; Wang et al., 2019; Mirmostafaee et al., 2020; Sekine et al., 2020; Werrie et al., 2020). However, the intensity and direction of plant responses are strongly influenced by donor material, extraction method, application dose, and recipient species. Low concentrations of plant-derived extracts may also induce slight stimulatory or compensatory responses, whereas higher concentrations often cause inhibition, showing a biphasic or hormetic pattern (Potters et al., 2007; Erofeeva, 2022; Wang et al., 2022; Abdelmalik et al., 2024; Liu et al., 2025; Siemieniuk et al., 2025; Solodka and Solodka, 2025).

A more cautious interpretation of dose-dependent phytotoxicity requires distinguishing cinnamon-specific effects from broader patterns of concentration-, species-, and endpoint-dependent plant responses. Similar interpretive issues have been discussed in other plant-stress contexts. For example, Li et al. (2023) used a meta-analysis of carbon-based nanomaterials to show that crop responses can shift from beneficial or compensatory effects to detrimental outcomes depending on species, dose, and measured endpoint. Although carbon-based nanomaterials differ fundamentally from allelopathic extracts, this framework is useful for interpreting multi-endpoint plant responses without over-attributing low-dose stimulation or species sensitivity to a single material-specific mechanism.

Root responses are especially relevant because roots are the first organ exposed to water-soluble phytotoxic substances in germination bioassays. Studies on underground plant interactions, root exudates, and rhizosphere communication suggest that recipient-plant responses depend not only on the presence of bioactive compounds but also on signal perception, oxidative-stress buffering, and tissue plasticity (Yoneyama and Bennett, 2024; Sorty et al., 2025; Bouwmeester et al., 2025; Zambelli et al., 2025). Therefore, evaluating cinnamon leaf aqueous extract solely by germination inhibition is insufficient for understanding the physiological basis of its phytotoxicity.

In this study, maize, a cereal crop, and lettuce, a model leafy vegetable crop, were selected as recipient species. Seed germination, seedling growth, antioxidant-enzyme activity, MDA content, chlorophyll content, root vigor, root-tip histology, multivariate statistics, and dose-response modeling were integrated. The novelty of the work lies in comparing two recipient species across multiple biological endpoints and in combining physiological-biochemical indicators with root-tip anatomical observations to characterize laboratory-scale phytotoxic responses. The study asked four questions: (1) whether cinnamon leaf aqueous extract produces concentration-associated phytotoxic responses; (2) whether the most responsive endpoints differ between maize and lettuce; (3) whether oxidative-stress-related and root-tip anatomical changes are associated with growth inhibition; and (4) whether model-derived effect ranges can provide preliminary guidance for future dose-selection experiments. These effect ranges are treated as laboratory-derived estimates rather than field-level ecological safety thresholds.

## 2 Materials and methods

### 2.1 Experimental materials

Cinnamon leaves were collected from mature cinnamon plants grown in the greenhouse of Guangdong Pharmaceutical University. After natural air drying, the leaves were ground and passed through a 60-mesh sieve. The maize variety was Zhengdan 958, and the lettuce variety was Italian bolting-resistant lettuce. Seeds were purchased from local seed companies. The seed-lot germination rate provided by the supplier or confirmed before the bioassay was ≥95%. This seed-lot value was used only as a quality criterion for selecting seeds; the germination rates reported in the Results represent actual germination under the present sterilization and Petri-dish bioassay conditions.

The main reagents included triphenyltetrazolium chloride (TTC), nitroblue tetrazolium (NBT), guaiacol, and thiobarbituric acid (TBA), all of analytical grade.

### 2.2 Preparation and definition of cinnamon leaf aqueous extract

One hundred grams of dry cinnamon leaf powder was extracted in 1000 mL distilled water at 80 °C for 4 h. After extraction, the mixture was filtered through qualitative filter paper and diluted with distilled water to a final volume of 1000 mL. This solution was defined as the 100% stock solution and corresponded to 100 g dry leaf equivalent L⁻¹. The stock solution was diluted with distilled water to obtain 1%, 10%, and 20% treatment solutions, corresponding to 1, 10, and 20 g dry leaf equivalent L⁻¹, respectively. Distilled water was used as the 0% control. The extract solutions were prepared before the germination assay and used on the same day.

### 2.3 Experimental design

Seed germination experiments were conducted using the Petri-dish filter-paper method. Plump and uniform seeds were selected, surface-sterilized with 0.3% NaOCl for 10 min, and rinsed five times with distilled water. Two layers of filter paper were placed in 9 cm Petri dishes, and 5 mL of the corresponding extract solution was added. Fifty seeds were sown in each dish, with three biological replicates per treatment. The culture conditions were 25 ± 1 °C, relative humidity of approximately 70%, light intensity of approximately 3000 lx, and a 12 h/12 h light/dark cycle. Culture was continued for 7 days, and the number of newly germinated seeds was recorded daily.

### 2.4 Measurement indicators

Germination rate (GR) was calculated as GR (%) = (number of germinated seeds/total number of seeds tested) × 100. Germination index (GI) was calculated as GI = Σ(Gt/t), where Gt is the number of newly germinated seeds on day t.

Seedling height and root length were measured after 7 days of culture. Root vigor was determined using the TTC reduction method (Towill and Mazur, 1975). Chlorophyll was extracted with ethanol and calculated according to Arnon (1949). SOD activity was determined using the NBT photochemical reduction method (Giannopolitis and Ries, 1977), POD activity using the guaiacol method (Chance and Maehly, 1955), and MDA content using the TBA method (Heath and Packer, 1968).

### 2.5 Root-tip histology and image quantification

Maize root tips from each treatment were fixed, dehydrated, embedded in paraffin, sectioned longitudinally, and stained with safranin-fast green. Representative root-tip images corresponding to 0%, 1%, 10%, and 20% treatments were used for anatomical comparison. Images were calibrated using the 100 μm scale bar before quantitative analysis.

For image-level quantification, five square regions of interest (ROIs) of identical size were selected from comparable positions in the proximal meristematic region of each representative microscopic image. The ROIs were chosen relative to the root cap-meristem boundary. Root cap tissue, central vascular/procambial columns, damaged margins, section folds, tears, and obvious staining artifacts were excluded. The descriptors were obtained from technical ROIs within representative images and should therefore be interpreted as image-level descriptive evidence rather than independent biological replication.

Four quantitative descriptors were calculated. Gap fraction was defined as the proportion of low-staining or empty-space pixels within an ROI after grayscale conversion and threshold segmentation. Edge density was defined as the proportion of edge pixels detected by an edge-detection algorithm relative to the total ROI pixel number. Texture entropy was calculated from the grayscale intensity histogram using Shannon entropy, H = -Σ pi log2(pi), where pi is the probability of gray level i. Staining index was calculated as mean(R) - mean(G), where mean(R) and mean(G) are the mean red- and green-channel intensities within the ROI; negative values indicate relatively stronger fast-green/cyan staining than safranin staining. Image processing was conducted using ImageJ/Fiji and Python-based image-analysis routines. The use of these image-level descriptors was adapted from previous quantitative plant histology and plant image-analysis approaches for root anatomical quantification, stained tissue segmentation, average coloration, and gray-level texture features (Lartaud et al., 2015; Legland et al., 2017, Legland et al., 2020; Lopez-Marnet et al., 2022).

### 2.6 Statistical analysis

All biological-replicate data were first subjected to Shapiro-Wilk normality tests and Levene homogeneity-of-variance tests, followed by one-way ANOVA and Duncan multiple comparisons at P < 0.05. Pearson correlation analysis and principal component analysis (PCA) were used to summarize relationships among traits (Jolliffe and Cadima, 2016). Statistical calculations were performed in SPSS 26.0 and Origin 2024.

Relative root length was used as the response variable for four-parameter logistic (4PL) fitting following the general dose-response modeling framework of Ritz et al. (2015). The model was y = Bottom + (Top - Bottom)/[1 + (x/EC50)^Hill slope^], where y is relative root length, x is extract concentration, Bottom and Top are the lower and upper asymptotes, EC50 is the midpoint concentration between Bottom and Top, and Hill slope indicates curve steepness. EC10, EC20, and EC50 were used only to describe model-derived effect ranges within the tested concentration interval.

## 3 Results

### 3.1 Effects of cinnamon leaf aqueous extract on lettuce

**Figure 1:**
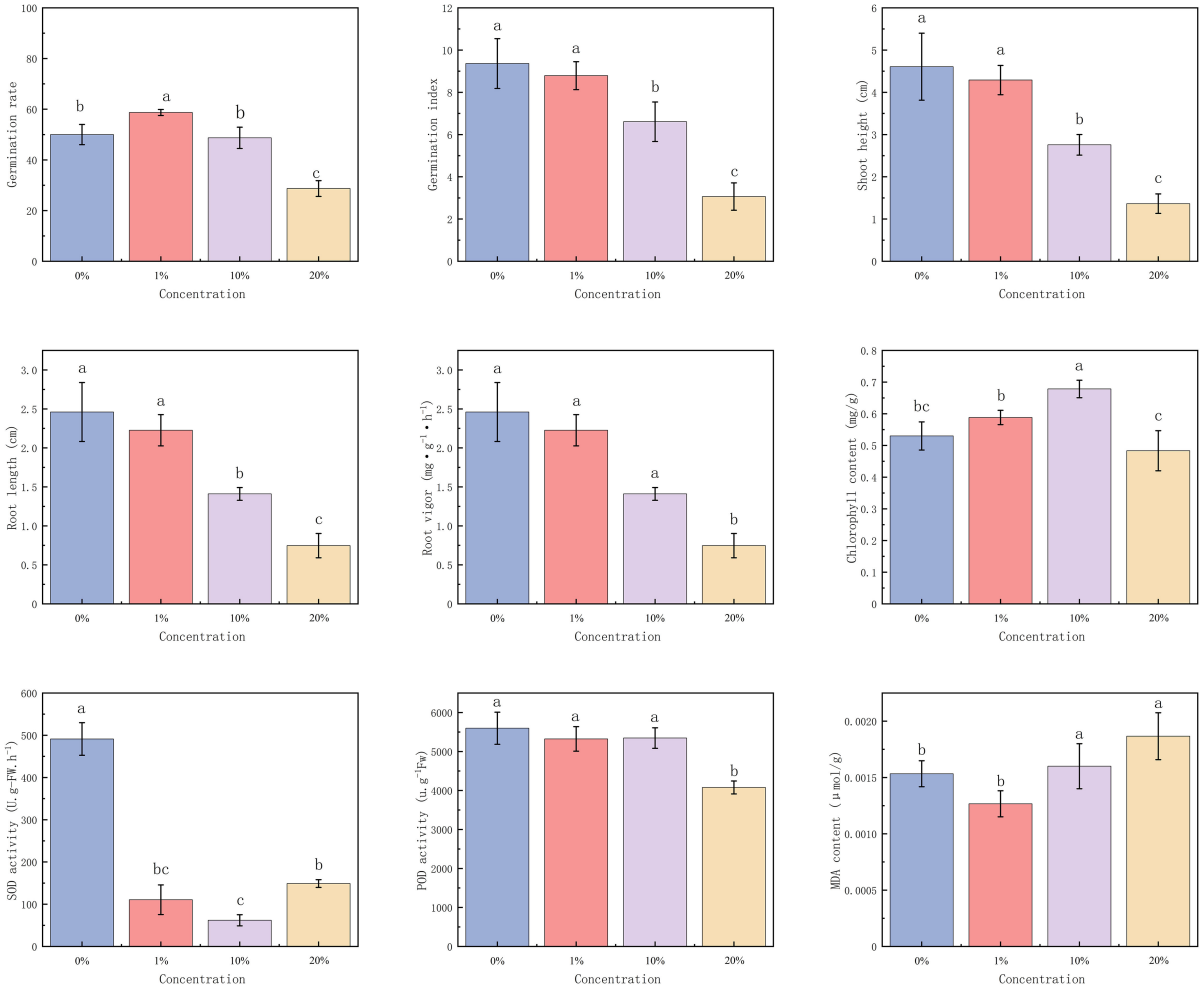
Effects of cinnamon leaf aqueous extract on germination, growth, and physiological-biochemical traits of lettuce (mean ± SD). Different lowercase letters indicate significant differences at P < 0.05. Germination rate is shown as percentage after unit correction.

Lettuce showed concentration-associated responses to cinnamon leaf aqueous extract (**Table 1**; **Figure 1**). The control, 1%, 10%, and 20% treatments had GR values of 50.0%, 58.7%, 48.7%, and 28.7%, respectively. The 1% treatment showed a slight increase relative to the control, whereas 20% markedly reduced germination and subsequent growth. Seedling height and root length also decreased at higher concentrations, with root length declining from 2.460 cm in the control to 0.747 cm under 20% treatment.

**Table 1:** Effects of cinnamon leaf aqueous extract on germination, growth, and physiological-biochemical traits of lettuce (mean ± SD, n = 3). .

Trait	0%	1%	10%	20%
Germination rate (GR, %)	50.0 ± 4.0 b	58.7 ± 1.2 a	48.7 ± 4.2 b	28.7 ± 3.1 c
Germination index (GI)	9.363 ± 1.179 a	8.790 ± 0.661 a	6.607 ± 0.936 b	3.063 ± 0.647 c
Shoot height (cm)	4.607 ± 0.794 a	4.290 ± 0.347 a	2.757 ± 0.243 b	1.363 ± 0.231 c
Root length (cm)	2.460 ± 0.378 a	2.227 ± 0.200 a	1.410 ± 0.082 b	0.747 ± 0.156 c
Root vigor (mg g⁻¹ h⁻¹)	0.033 ± 0.006 a	0.033 ± 0.004 a	0.028 ± 0.001 a	0.018 ± 0.002 b
Chlorophyll content (mg g⁻¹)	0.530 ± 0.044 bc	0.588 ± 0.023 b	0.678 ± 0.028 a	0.483 ± 0.063 c
SOD activity (U g⁻¹ FW h⁻¹)	491.150 ± 38.576 a	110.620 ± 35.119 bc	61.947 ± 13.275 c	148.967 ± 9.211 b
POD activity (U g⁻¹ FW)	5596.877 ± 412.084 a	5323.127 ± 314.814 a	5345.627 ± 263.254 a	4078.127 ± 166.202 b
MDA content (μmol g⁻¹)	0.0015 ± 0.0001 b	0.0013 ± 0.0001 b	0.0016 ± 0.0002 a	0.0019 ± 0.0002 a

Different lowercase letters within a row indicate significant differences at P < 0.05 according to Duncan multiple range test. GR is expressed as percentage. DLE, dry leaf equivalent.

At the physiological level, lettuce SOD activity showed a non-linear response rather than a strictly monotonic decline. Compared with the control, SOD activity decreased sharply at 1% and 10%, reached the lowest value at 10%, and then partially rebounded at 20%, although it remained far below the control. POD activity decreased significantly at 20%, whereas MDA content increased under higher concentrations. These results indicate that lettuce responses involved oxidative-stress-related changes and membrane lipid peroxidation, but the antioxidant response was not a simple concentration-dependent collapse.

### 3.2 Effects of cinnamon leaf aqueous extract on maize

**Figure 2:**
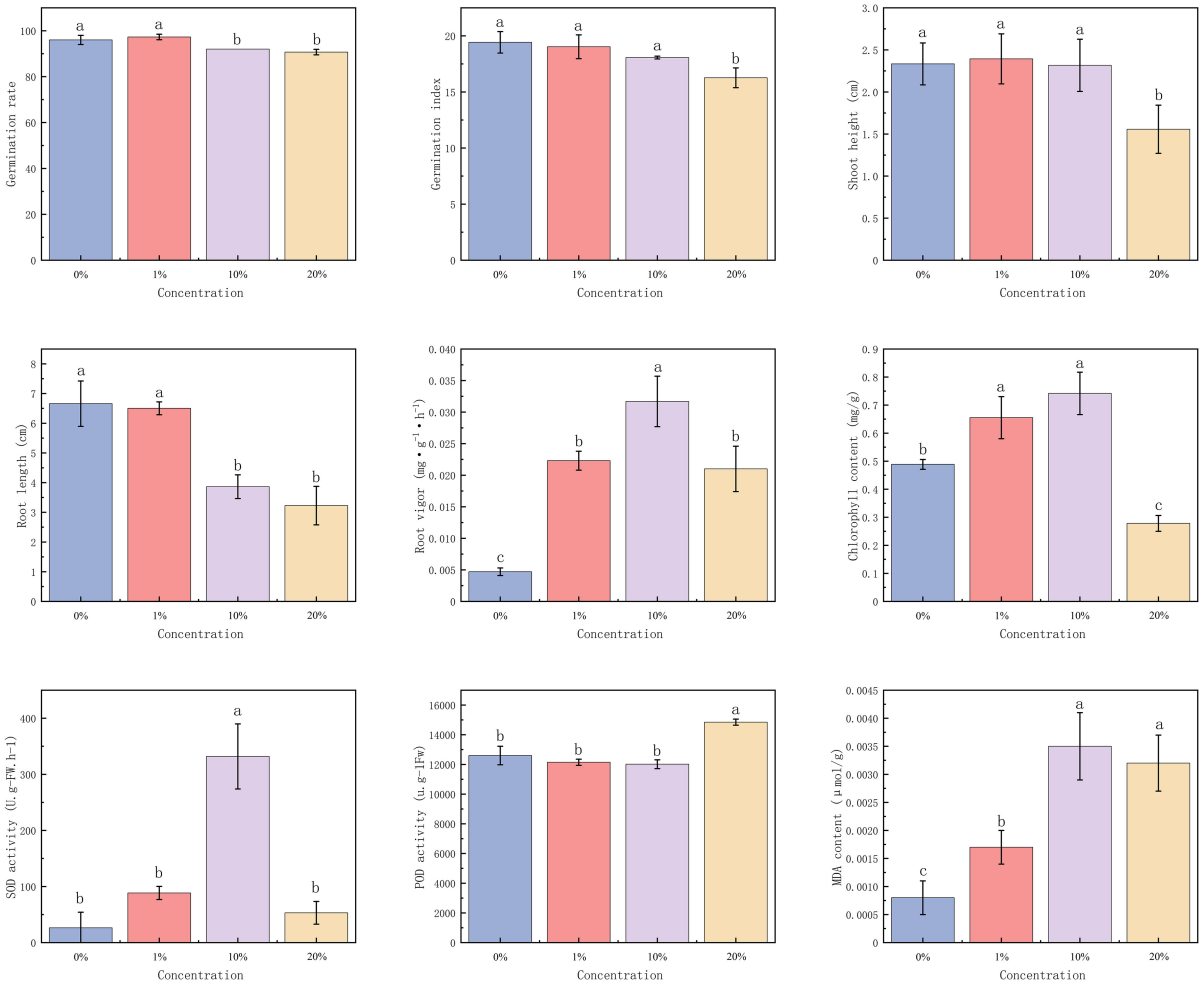
Effects of cinnamon leaf aqueous extract on germination, growth, and physiological-biochemical traits of maize (mean ± SD). Different lowercase letters indicate significant differences at P < 0.05. Germination rate is shown as percentage after unit correction.

Maize showed stronger tolerance than lettuce during the final germination stage (**Table 2**; **Figure 2**). After correction of GR units, maize germination remained above 90% across all treatments. However, post-germination root elongation was more sensitive to the extract. Relative to the control, root length decreased by approximately 42.0% and 51.5% under 10% and 20% treatments, respectively. The 1% treatment did not significantly inhibit seedling height or root length.

**Table 2:** Effects of cinnamon leaf aqueous extract on germination, growth, and physiological-biochemical traits of maize (mean ± SD, n = 3).

Trait	0%	1%	10%	20%
Germination rate (GR, %)	96.0 ± 2.0 a	97.3 ± 1.2 a	92.0 ± 0.0 b	90.7 ± 1.2 b
Germination index (GI)	19.427 ± 0.959 a	19.030 ± 1.058 a	18.070 ± 0.125 a	16.263 ± 0.881 b
Shoot height (cm)	2.333 ± 0.249 a	2.393 ± 0.297 a	2.317 ± 0.310 a	1.557 ± 0.286 b
Root length (cm)	6.660 ± 0.764 a	6.503 ± 0.217 a	3.863 ± 0.400 b	3.227 ± 0.647 b
Root vigor (mg g⁻¹ h⁻¹)	0.005 ± 0.001 c	0.022 ± 0.002 b	0.032 ± 0.004 a	0.021 ± 0.004 b
Chlorophyll content (mg g⁻¹)	0.489 ± 0.017 b	0.655 ± 0.075 a	0.742 ± 0.076 a	0.278 ± 0.028 c
SOD activity (U g⁻¹ FW h⁻¹)	26.357 ± 27.863 b	88.497 ± 11.709 b	331.860 ± 58.033 a	53.097 ± 20.280 b
POD activity (U g⁻¹ FW)	12601.880 ± 624.586 b	12146.253 ± 207.620 b	12015.000 ± 298.284 b	14848.127 ± 206.392 a
MDA content (μmol g⁻¹)	0.0008 ± 0.0003 c	0.0017 ± 0.0003 b	0.0035 ± 0.0006 a	0.0032 ± 0.0005 a

Different lowercase letters within a row indicate significant differences at P < 0.05 according to Duncan multiple range test. GR is expressed as percentage. DLE, dry leaf equivalent.

The maize physiological response differed from that of lettuce. MDA content increased strongly under 10% and 20% treatments. SOD activity peaked at 10%, POD activity increased significantly under 20%, and root vigor was higher under extract treatments than in the control. These patterns suggest that maize roots activated compensatory or stress-buffering responses under some treatment conditions, although these responses were insufficient to maintain normal root elongation at higher concentrations.

### 3.3 Daily cumulative germination dynamics

**Figure 3:**
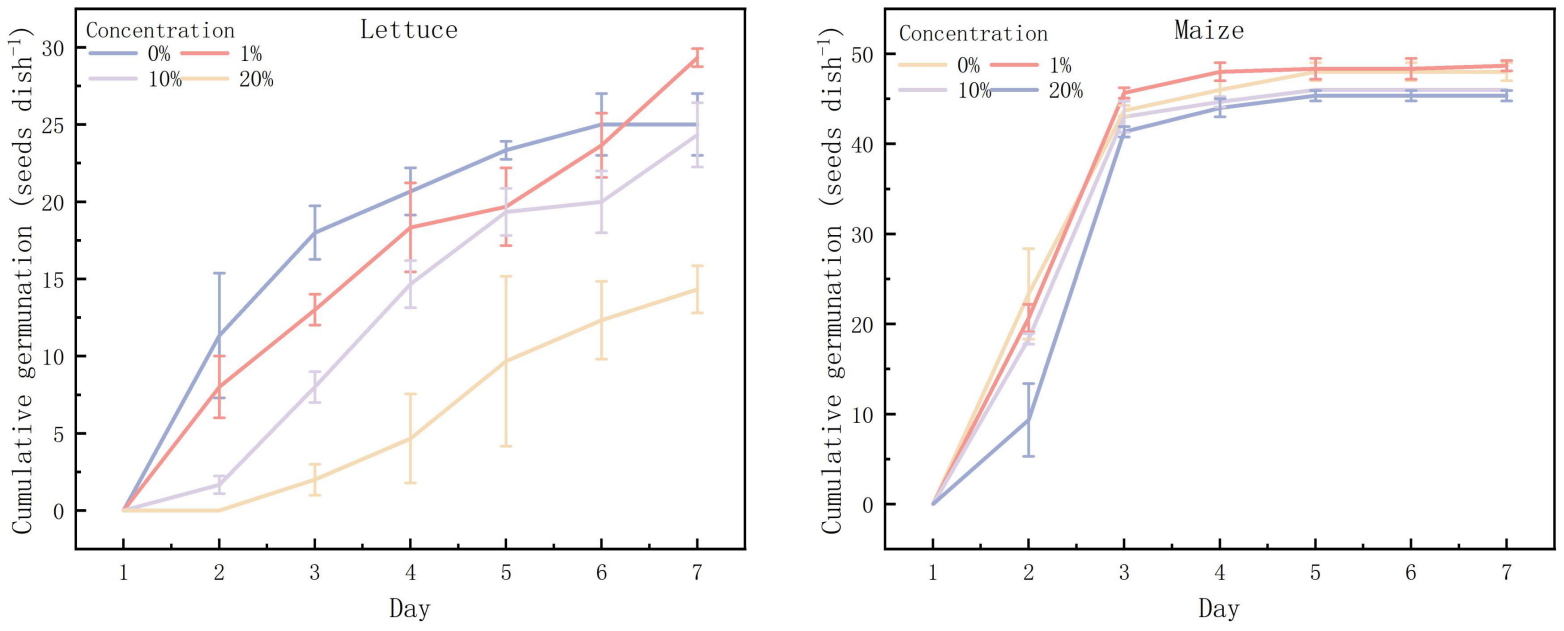
Daily cumulative germination dynamics of lettuce and maize under cinnamon leaf extract treatments (mean ± SD).

The daily cumulative germination curves revealed temporal differences between the two recipient plants (**Figure 3**). Lettuce showed delayed and reduced cumulative germination under 10% and 20% treatments. In maize, the final germination rate tended to remain high, but the 20% treatment delayed early germination initiation. Thus, lettuce was more responsive in germination dynamics, whereas maize showed stronger post-germination root-length sensitivity.

### 3.4 Prerequisite tests, correlation analysis, PCA, and dose-response modeling

**Figure 4:**
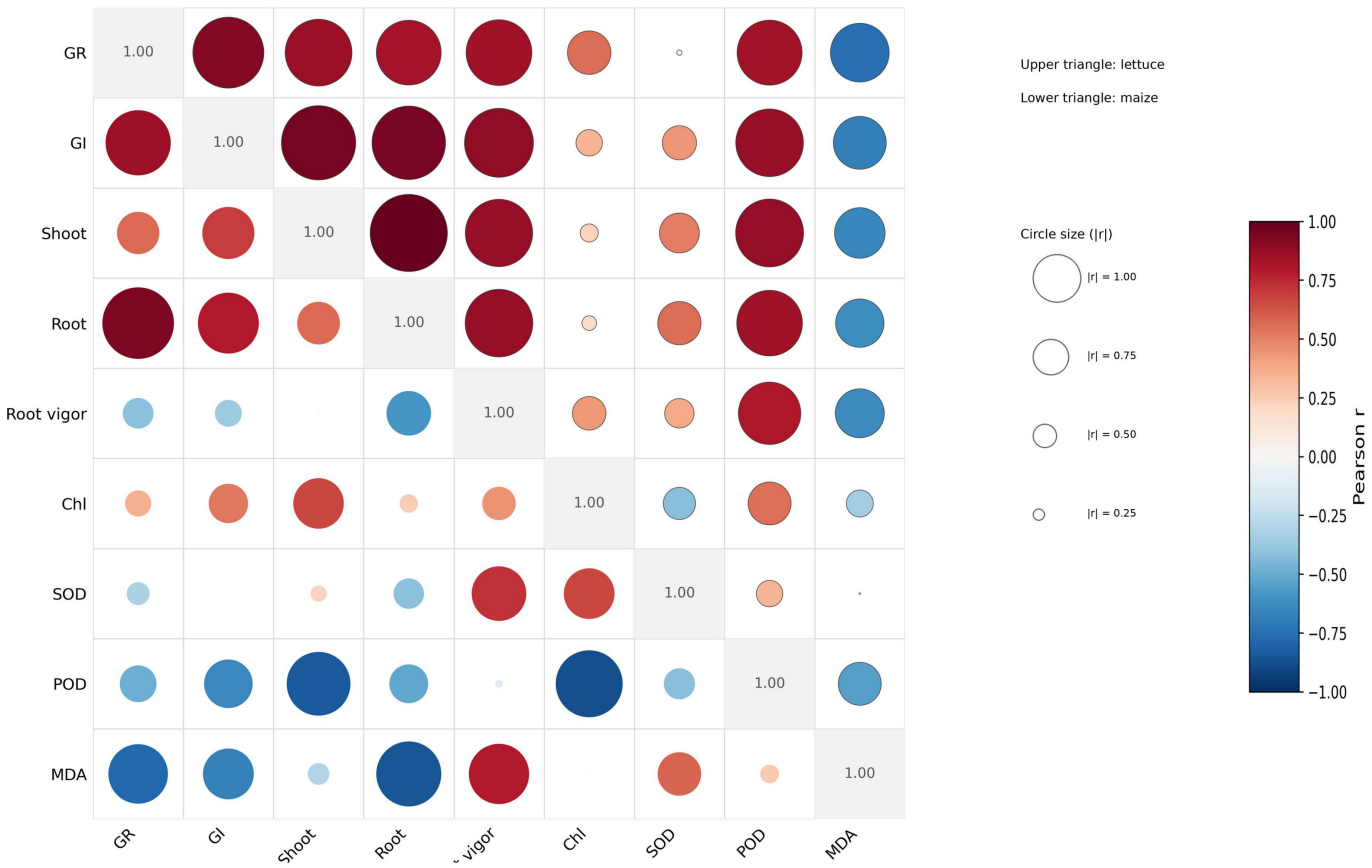
Combined circular correlation heatmap of physiological-biochemical traits in lettuce and maize. The upper triangle represents lettuce and the lower triangle represents maize; circle size indicates |r| and color indicates the direction and magnitude of correlation.

**Figure 5:**
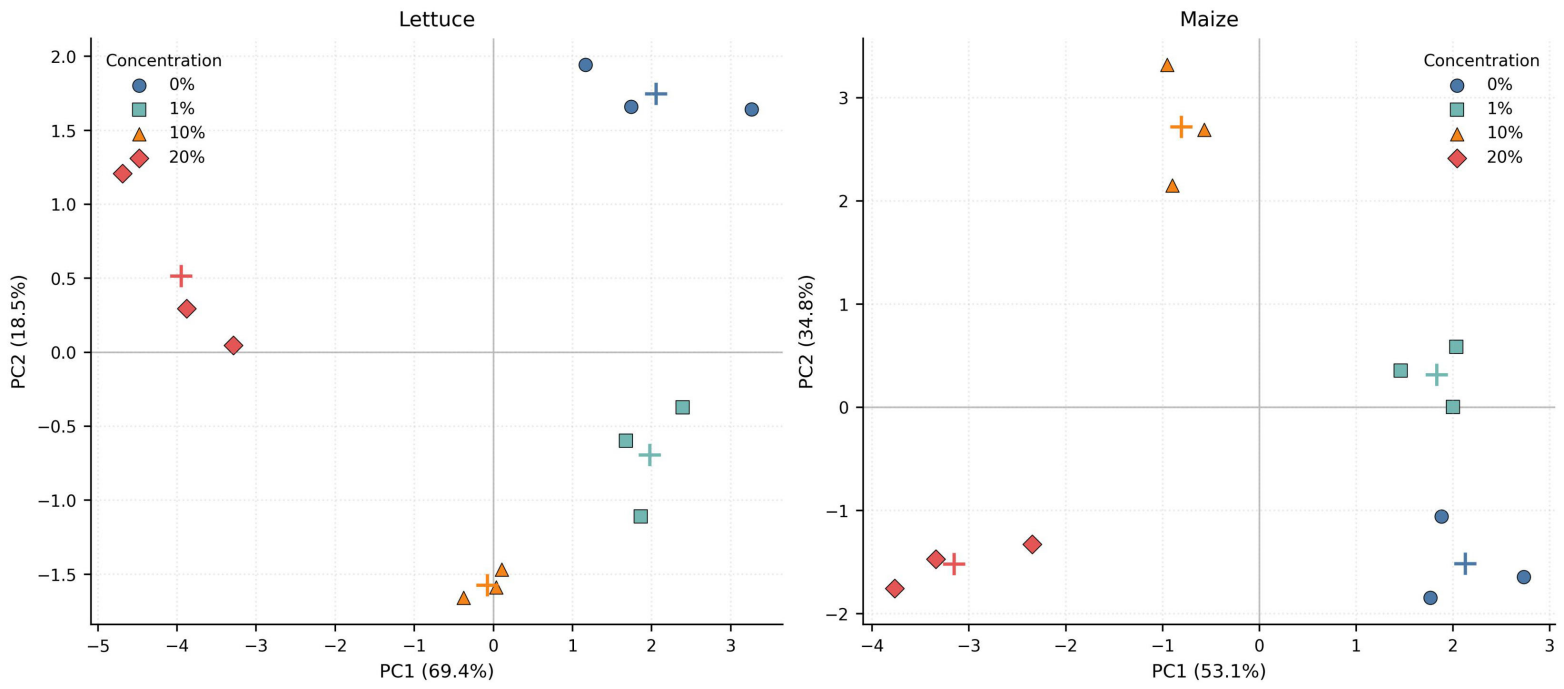
PCA score plot of lettuce and maize samples under different concentrations of cinnamon leaf aqueous extract.

Most measured traits met the prerequisites for one-way ANOVA (**Table 3**). Correlation analysis showed positive relationships among GR, GI, shoot height, and root length, whereas MDA was generally negatively associated with growth indicators (**Figure 4**). PCA separated low- and high-concentration treatments along the first principal component (**Figure 5**), indicating coordinated shifts from high-growth/low-damage states toward low-growth/high-stress states.

**Table 3:** Shapiro-Wilk normality test, Levene homogeneity test, and one-way ANOVA results for measured traits.

Species	Trait	Shapiro P	Levene P	F	P
Lettuce	Germination rate (GR)	0.5487	0.6722	43.909	<0.0001
Lettuce	Germination index (GI)	0.7789	0.8253	31.294	<0.0001
Lettuce	Shoot height	0.8131	0.4016	31.181	<0.0001
Lettuce	Root length	0.7632	0.6562	34.537	<0.0001
Lettuce	Root vigor	0.2771	0.6415	12.377	0.0023
Lettuce	Chlorophyll content	0.9520	0.6785	11.687	0.0027
Lettuce	SOD activity	0.9999	0.6970	153.401	<0.0001
Lettuce	POD activity	0.6234	0.8393	15.312	0.0011
Lettuce	MDA content	0.6414	0.8380	6.626	0.0146
Maize	Germination rate (GR)	0.9362	0.4872	18.133	0.0006
Maize	Germination index (GI)	0.7905	0.7549	8.424	0.0074
Maize	Shoot height	0.2007	0.9961	5.756	0.0214
Maize	Root length	0.7108	0.6484	31.181	<0.0001
Maize	Root vigor	0.9781	0.6926	47.205	<0.0001
Maize	Chlorophyll content	0.7676	0.6474	40.170	<0.0001
Maize	SOD activity	0.8870	0.3985	50.313	<0.0001
Maize	POD activity	0.9882	0.4221	37.079	<0.0001
Maize	MDA content	0.1760	0.8251	26.944	0.0002

**Figure 6:**
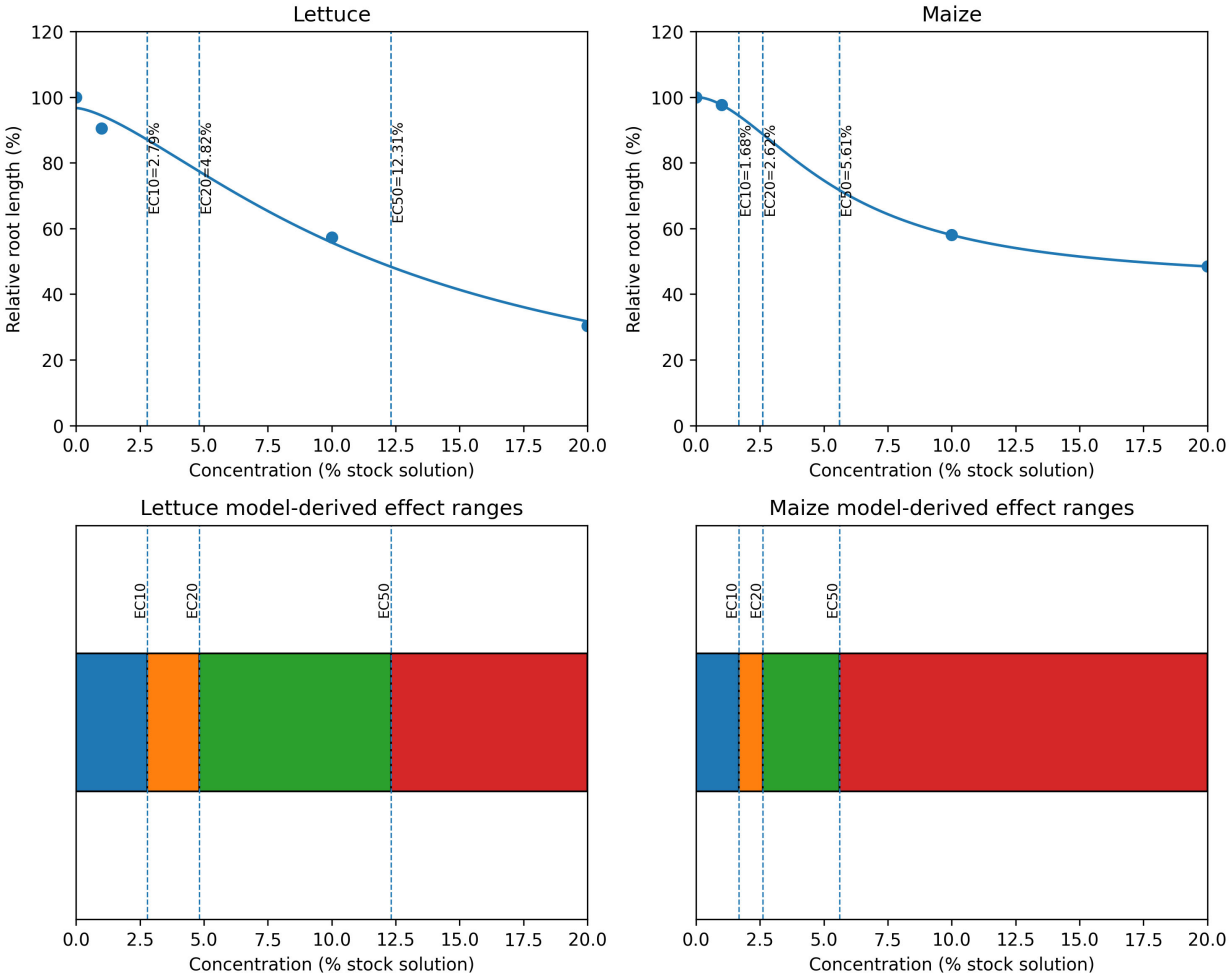
Dose-response fitting and model-derived effect ranges for lettuce and maize. The upper panels show 4PL curves based on relative root length, and the lower panels show concentration ranges defined by EC10, EC20, and EC50. Ranges are restricted to the tested interval of 0-20% and should not be interpreted as field-level ecological safety thresholds.

The 4PL model based on relative root length estimated EC50 values of 12.31% for lettuce and 5.61% for maize (**Table 4**; **Figure 6**). However, since the experiment only included treatment levels of 0%,1%,10%, and 20%, these values should be interpreted as preliminary model-derived estimates and are confined to the measured range of 0–20% (**Table 5**).

**Table 4:** Four-parameter logistic (4PL) model parameters fitted using relative root length as the response variable.

Species	Bottom	Top	EC10 (%)	Approx. 95% CI for EC10	EC20 (%)	Approx. 95% CI for EC20	EC50 (%)	95% CI for EC50	Hill slope	R²	RMSE (%)
Lettuce	0.000	96.693	2.787	1.534-3.217	4.822	2.654-5.567	12.311	6.777-14.213	1.479	0.990	2.747
Maize	43.356	100.000	1.677	0.509-3.806	2.618	0.795-5.942	5.608	1.702-12.728	1.820	>0.999	0.004

Confidence intervals for EC10 and EC20 are approximate transformations based on the EC50 confidence interval and fixed Hill slope; all thresholds should be interpreted cautiously because only four nominal concentrations were tested.

**Table 5:** Model-derived effect ranges defined by EC10, EC20, and EC50 values.

Species	Model-derived effect range	Lower (%)	Upper (%)
Lettuce	Minimal-effect range	0.000	2.787
Lettuce	Low-effect range	2.787	4.822
Lettuce	Intermediate-effect range	4.822	12.311
Lettuce	High-effect range	12.311	20.000
Maize	Minimal-effect range	0.000	1.677
Maize	Low-effect range	1.677	2.618
Maize	Intermediate-effect range	2.618	5.608
Maize	High-effect range	5.608	20.000

The ranges are restricted to the tested concentration interval of 0-20% and should not be interpreted as field-level ecological safety thresholds.

### 3.5 Root-tip anatomical response

**Figure 7:**
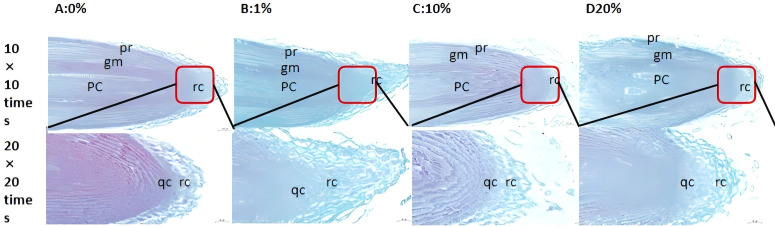
Safranin-fast green-stained paraffin sections of maize root tips. **(A)**, 0%; **(B)**, 1%; **(C)**, 10%; **(D)**, 20%. pr, protoderm/peripheral region; gm, ground meristem; pc, procambium; rc, root cap; qc, quiescent center. Scale bars: 100 μm in low-magnification panels and 50 μm in enlarged panels.

Safranin-fast green sections showed visible structural differences in maize root tips among treatments (**Figure 7**). In the control and 1% treatment, cell columns were relatively continuous and peripheral meristematic tissues were compact. In the 10% and 20% treatments, peripheral tissues appeared looser, cell-row continuity decreased, and the boundary between the root cap and meristematic region became less distinct.

**Figure 8:**
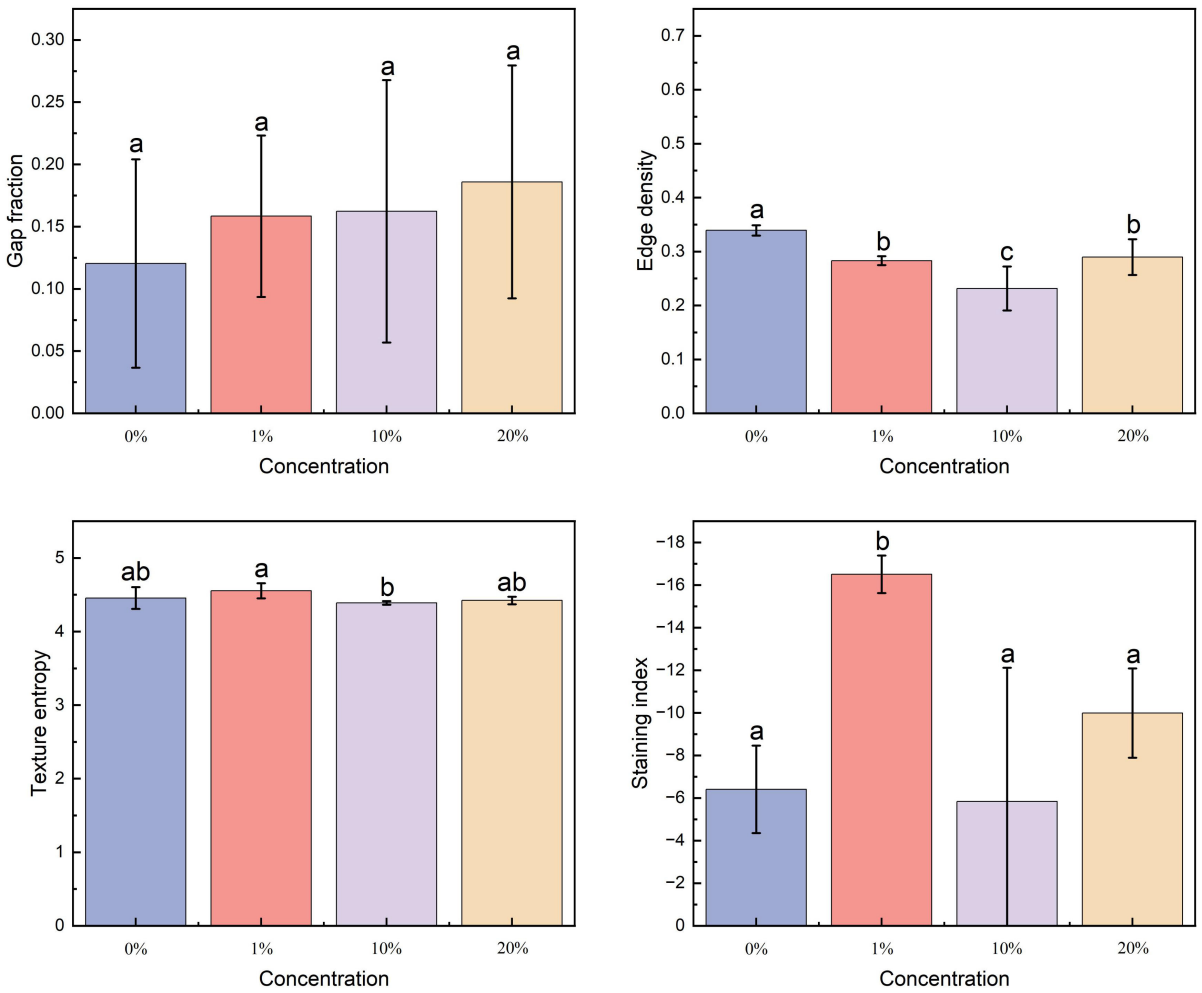
Error-bar plots of quantitative descriptors extracted from maize root-tip meristem images. The descriptors are based on technical ROIs from representative images and provide image-level descriptive evidence.

ROI-based descriptors provided image-level quantitative support for the anatomical observations (**Table 6**; **Figure 8**). Gap fraction tended to increase under higher concentrations, and edge density, texture entropy, and staining index varied among treatments. Because these descriptors were calculated from five technical ROIs selected from representative images, they should be interpreted as descriptive image-level evidence rather than independent biological replication.

**Table 6:** Quantitative image descriptors of maize root-tip meristem ROIs (mean ± SD, n = 5 technical ROIs).

Descriptor	0%	1%	10%	20%
Gap fraction	0.120 ± 0.084 a	0.158 ± 0.065 a	0.162 ± 0.105 a	0.186 ± 0.094 a
Edge density	0.339 ± 0.009 a	0.283 ± 0.008 b	0.232 ± 0.041 c	0.290 ± 0.033 b
Texture entropy	4.455 ± 0.148 ab	4.555 ± 0.103 a	4.390 ± 0.026 b	4.422 ± 0.051 ab
Staining index	-6.408 ± 2.057 a	-16.510 ± 0.883 b	-5.833 ± 6.288 a	-9.990 ± 2.093 a

This analysis was based on representative paraffin-section images and should be interpreted as image-level descriptive evidence rather than independent biological replication.

## 4 Discussion

The present study showed that cinnamon leaf aqueous extract was associated with concentration-dependent phytotoxic responses in both maize and lettuce. However, the most responsive biological endpoints differed between species. Lettuce showed greater sensitivity in germination and whole-seedling growth, whereas maize maintained high final germination but exhibited strong inhibition of root elongation after germination. This separation between germination sensitivity and post-germination root-growth sensitivity supports the broader view that plant responses to phytotoxic materials are dose-, species-, and endpoint-dependent (Weston and Duke, 2003; Weir et al., 2004; Khamare et al., 2022; Kumar et al., 2024).

The low-dose stimulation observed for some traits should be interpreted cautiously. It is consistent with hormesis-like or compensatory plant responses under mild stress, but it does not by itself demonstrate a cinnamon-specific mechanism. Similar multi-endpoint response frameworks have been described outside allelopathy. For instance, Li et al. (2023) showed that carbon-based nanomaterials can induce both beneficial and detrimental crop responses depending on concentration, species, and endpoint. By analogy, the present findings suggest that cinnamon leaf aqueous extract should be evaluated using integrated endpoints rather than a single germination-inhibition value.

Changes in MDA, SOD, POD, and root vigor indicate that extract treatments were associated with oxidative-stress-related responses and membrane lipid peroxidation. Nevertheless, the present data do not directly measure ROS accumulation and cannot establish a strict causal sequence from oxidative imbalance to meristematic disruption and growth inhibition. Therefore, the proposed pathway should be viewed as a mechanistic hypothesis supported by associated physiological and anatomical patterns. The maize results further demonstrate this complexity: increased SOD activity, POD activity, and root vigor under some treatments suggest compensatory antioxidant or dehydrogenase responses rather than a uniform oxidative-collapse mechanism (Gill and Tuteja, 2010; Sharma et al., 2012; Mittler et al., 2022; Rao et al., 2025; Alam et al., 2025).

The root-tip histological observations provide structural context for the physiological data. Under higher extract concentrations, the maize meristematic zone showed looser peripheral tissues and reduced cell-row continuity. These observations are consistent with growth inhibition and altered oxidative-stress-related status, similar to root hair inhibition and ROS imbalance reported in radish seedlings treated with rhizome extracts of invasive Fallopia species (Šoln et al., 2025). However, because ROI quantification was based on technical ROIs from representative images, the results should not be interpreted as independent biological replication. Future work should analyze multiple independent seedlings, root tips, serial sections, and microscopic fields per treatment to support stronger statistical inference. Similar image-based approaches have been used to quantify plant tissue sections, including root anatomical traits, stained tissue regions, average coloration, and gray-level texture features; therefore, the present ROI analysis should be interpreted as descriptive image-level support for anatomical observations rather than as independent histological replication (Lartaud et al., 2015; Legland et al., 2017, Legland et al., 2020; Lopez-Marnet et al., 2022).

The dose-response modeling also requires cautious interpretation. Although the 4PL model summarized the decline in relative root length, only four nominal concentrations were tested, with a large gap between 1% and 10%. The model-derived EC50 values for maize and lettuce fell in intervals without intermediate experimental data. Therefore, the EC10, EC20, and EC50 values are preliminary laboratory estimates, not experimentally validated safety thresholds. Additional intermediate concentrations, such as 2.5%, 5%, 7.5%, 12.5%, and 15%, should be included in future experiments to improve model precision and confidence-interval estimation.

Another limitation is the chemical definition of the extract. In this study, extract concentration was expressed as dry leaf equivalent and stock-solution dilution, but the chemical composition was not profiled, and pH and electrical conductivity were not measured. Therefore, the observed effects should be attributed to the aqueous extract as a whole rather than to specific cinnamon-derived allelochemicals. Chemical characterization, including phenolic and volatile profiles as well as pH and EC measurements, will be necessary to identify active fractions and distinguish chemical toxicity from general solution effects.

Finally, ecological and application implications should be bounded by the laboratory nature of the experiment. Petri-dish bioassays are useful for comparative phytotoxicity screening, but they do not establish field-level ecological safety or practical herbicidal applicability. In soil systems, adsorption, dilution, biodegradation, rhizosphere processes, microbial transformation, environmental variability, and non-target effects may substantially alter the availability, persistence, and biological activity of plant-derived extracts. Recent work on environmental-risk assessment has emphasized the value of integrating multiple environmental drivers when evaluating complex ecological hazards (Li et al., 2026). Although that study addressed antibiotic-resistance genes in the plastisphere rather than allelopathy, it provides a useful conceptual example for why cinnamon-derived materials should be assessed under multi-factor environmental conditions before practical application.

## 5 Conclusion

Cinnamon leaf aqueous extract induced concentration-associated phytotoxic responses in maize and lettuce under laboratory Petri-dish conditions. Lettuce was more responsive in germination and whole-seedling growth, whereas maize showed stronger sensitivity in post-germination root elongation. High extract concentrations were associated with increased MDA, altered antioxidant-enzyme activities, and visible changes in maize root-tip meristem organization. These physiological and anatomical patterns are consistent with a possible pathway involving oxidative-stress-related responses, membrane lipid peroxidation, meristematic disturbance, and growth inhibition, but they do not directly establish a causal mechanism.

Model-derived EC10, EC20, and EC50 values based on relative root length provide preliminary effect-range estimates within the tested laboratory system. Because the concentration gradient was coarse, the extract was not chemically characterized, and the histological ROI analysis was image-level descriptive evidence, further experiments with intermediate concentrations, chemical profiling, independent histological replication, soil-based assays, and non-target evaluations are required before ecological safety or practical application can be concluded.

## Data Availability

The original contributions presented in the study are included in the article/supplementary material, further inquiries can be directed to the corresponding author/s.
